# Public Attitudes Toward Priority Setting Principles in Health Care During COVID-19

**DOI:** 10.3389/frhs.2022.886508

**Published:** 2022-05-13

**Authors:** Gustav Tinghög, Liam Strand

**Affiliations:** ^1^Swedish National Centre for Priority Setting in Health Care, Department of Health, Medicine, and Caring Sciences, Linköping University, Linköping, Sweden; ^2^Department of Management and Engineering, Linköping University, Linköping, Sweden

**Keywords:** cost-effectiveness, health care priority setting, public attitude, Sweden, health care, medical ethics, health economics, rationing

## Abstract

What role should cost-effectiveness play in health care priority setting? We assess the level of acceptance toward different priority setting principles in health care during COVID-19 and in general, thereby exploring public support for principles presented at different levels of abstraction. An online survey was distributed to a diverse sample of the Swedish population (*n* = 1 553). The results show that respondents were generally more supportive of priority setting principles when expressed in general abstract terms than when expressed in more case specific concrete terms. However, prioritization based on cost-effectiveness was deemed as more acceptable when expressed in concrete terms related to health maximization rather than as an abstract principle. Respondents had a general inclination in support of physicians and other health care professionals the primary responsibility for the allocation of scarce resources in the healthcare during COVID-19, while being less supportive of health economists and politicians being involved in these decisions.

## Introduction

Health economics is often thought of as inhumane, promoting efficiency at the expense of more profound moral values such as equality and need. The fact that allocations solely based on cost-effectiveness are unlikely to be compatible with public views has been illustrated in experimental studies (1–3). Moreover, lessons from the Oregon experience on priority setting illuminated that rationing decisions, based on health maximization, are likely to conflict with the view of the general public (4, 5). For an economist, this conflict can be hard to understand. Why does not the quest to maximize the value for money strike a chord with the general public?

One possible explanation lies in the psychological phenomenon of how emotions make it difficult to feel magnitude. The quotes, by Mother Teresa “If I look at the mass I will never act. If I look at the one, I will”, and Joseph Stalin “One death is a tragedy; one million is a statistic” capture an essential aspect of human cognition and the role of emotions. These quotes give us a clue for why public views often are incompatible with the logics of economic thinking when it comes to health care priority setting. A fundamental assumption within health economics is that more is better than less. Saving two lives is better than saving 1 life; Gaining 10 Quality Adjusted Life years (QALYs) is better than gaining 9 QALYs, *ceteris paribus*. According to the rationale of health economics, the aggregate consequences should guide priority setting. Although most people agree with this general idea when contemplating about it on an abstract level (6, 7), a different story emerges when looking at real behavior. Numerous studies have shown that as the number of people in need increases, the degree of compassion people feel for them ironically tends to decrease (8, 9). This phenomenon has been termed the compassion fade (10). This compassion fade happens because emotions are not triggered by aggregates. “Statistical victims” at the aggregate level fail to spark emotion and compassion, while many feel strong compassion toward the single victim.

The issues of “statistical victims” and compassion fade are highly central to debates of health care priority setting. Health economic considerations and policy decisions typically concern statistical patients at the aggregate level, public debate and ethical discussions typically revolve around individual cases. Individuals (both those with and those without clinical experience) are more likely to support rationing health care presented at a general policy level compared to when presented with the equivalent decision at the bedside (11, 12). However, the effect of identifiability in the context of health care priority setting is not always positive for the identified patient (3, 13). This suggest that it is not only emotional valence that affect public views for how health care resources should be allocated.

Construal Level Theory (14) provides an additional potential psychological mechanism for why public views and policy decisions differ on the issue of rationing health care. This theory predicts that judgments are affected by the levels of construal representation (abstract or concrete). Higher-level abstraction prompts people to focus on central values instead of practical concerns. Studies have found that when policies describe specific outcomes, people become more oriented toward efficiency concerns, while they gravitate toward equality concerns when policies on resource allocation are described in more general terms (7, 15, 16). Such findings suggest that the level of abstraction used to describe priority setting principles may prompt emphasis on different values. The objective in the current study was to assess public attitudes toward different priority setting principles in health care during, described at different levels of abstraction. Moreover, we explored public attitudes toward whom should have the primary responsibility for making priority setting decisions regarding scarce health care resources during a global public health crisis like COVID-19.

## Methods

We conducted a survey to explore the public attitudes toward different priority setting principles in healthcare. The data was collected through an online survey from a subject pool with Swedish adults who had voluntarily enlisted themselves as being interested in participating in future studies after using a voting advice application by the Swedish newspaper Aftonbladet. The subject pool consist of 4 177 active subjects (34.4% females) and has been used in previous studies (17–19) and has previously been shown to accurately predict the Swedish general election of 2018 (20). The data collection was made during late August and early September 2020 and the sample consists of 1 553 participants. Participants had to be at least 18 years old. Mean age of the sample was 56.53 years old (SD = 14.59) and 65% were male. Approximately half of the subjects had at least three additional years of studies after secondary school (50%), 25% had at most 2 years of studies after secondary school, 18 % had at most secondary school, while the remaining 8% had at most completed primary school.

Table 1 show the statements included to measure public attitudes in the survey. Respondents were asked to express to what extent they agreed or disagreed with each presented statement using a 7-point Likert scale. The survey covered three main topics: (i) priority- setting principles of intensive care during COVID-19; (ii) general priority setting principles for healthcare; (iii) decision-maker for the healthcare's prioritizations during COVID-19. The order of statements was randomized within each topic, but the order of topics was fixed following the order in which they are presented in Table 1. To measure the participants attitudes toward priority-setting principles at intensive care during COVID-19, we used 10 statements that described principles in concrete terms (i.e., “Higher priority should be given to infected patients who have followed the Public Health Agency of Sweden's recommendations and acted responsibly to avoid the virus”). We chose to include concrete principles which has been commonly identified and discussed in the general literature on health care priority setting but adapted for COVID-19, including those that relate to both medical conditions and economic concerns (21–25). To measure attitudes toward general principles for healthcare prioritizations, we used 3 statements phrased as descriptions of the three lexically ordered principles that constitutes the legislated Swedish ethical platform for prioritization (26); the human dignity-, the needs and solidarity-, and the cost-effectiveness principle. Finally, to measure the attitudes toward who should primarily decide how to allocate the healthcare's resources during the COVID-19 pandemic, we asked respondents about the suitability of six groups or professions involved in priority setting.

**Table 1:** Statements in the Survey.

**Item**	
1,1	Higher priority should be given to infected patients who have followed the Public Health Agency of Sweden's recommendations and acted responsibly to avoid the virus.
1,2	Higher priority should be given to infected patients who have responsibilities to take care of children or other relatives.
1,3	Higher priority should be given to infected patients who have an important societal function.
1,4	Higher priority should be given to younger infected patients.
1,5	Higher priority should be given to infected patients with worse health conditions.
1,6	Higher priority should be given to infected patients where treatment is cost-effective
1,7	Higher priority should be given to infected patients where a limited effort is expected to have a large effect
1,8	If the resources are not sufficient to give everyone treatments it is reasonable to use a lottery to decide who should get treatment
1,9	If the resources are not sufficient to give everyone treatments it is reasonable to let the patient who has waited longest get treatment.
1,10	When you give treatment to an infected patient you should always consider what you instead could have used the resources for.
2,1	Human dignity principle: all humans have the same value and the same right to care independent of personal traits and function in society.
2,2	The needs- and solidarity principle: more of the healthcare's resources should be spent on the person or the organization which have the greatest needs.
2,3	The cost-effectiveness principle: in choices between different areas of operations or measures should a reasonable relation between costs and effects, measured in improved health or increased quality of life, be sought.
3,1	Decisions of how scarce resources in the healthcare should be allocated should primarily be decided by health economists.
3,2	Decisions of how scarce resources in the healthcare should be allocated should primarily be decided by politicians.
3,3	Decisions of how scarce resources in the healthcare should be allocated should primarily be decided by the general population.
3,4	Decisions of how scarce resources in the healthcare should be allocated should primarily be decided by ethicists
3,5	Decisions of how scarce resources in the healthcare should be allocated should primarily be decided by relatives
3,6	Decisions of how scarce resources in the healthcare should be allocated should primarily be decided by physicians and others within the medical profession.

## Results

**Figure 1:**
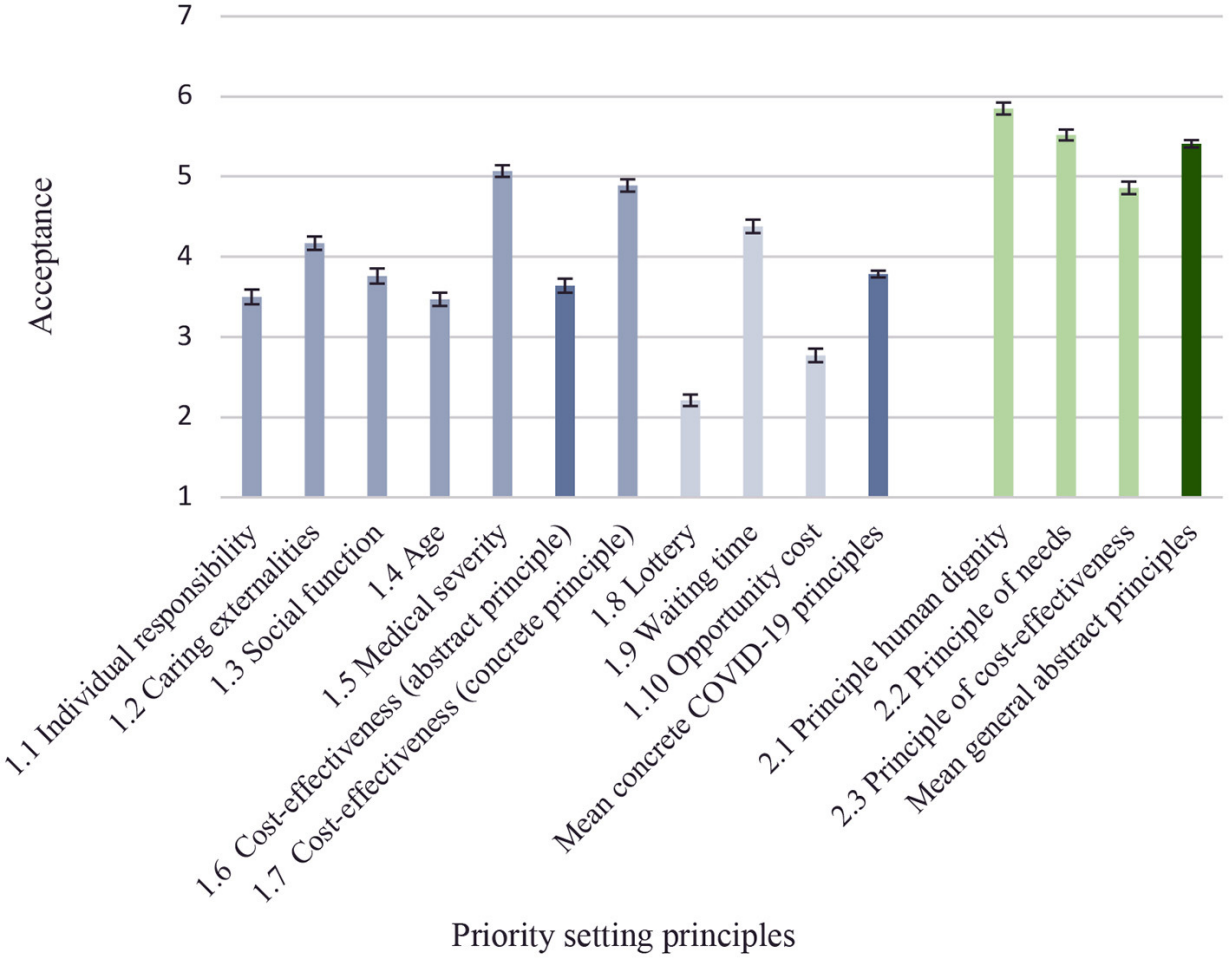
Attitudes toward priority-setting principles. The figure shows mean ratings of acceptance for each priority setting principle with bars for 95% confidence intervals. 1 = lowest acceptance, 7 = highest acceptance.

Figure 1 illustrates level of acceptance toward different priority setting principles. Looking at the more concrete principles in the context of COVID-19, the highest level of acceptance was found for prioritizing based on severity of the medical condition (mean acceptance rating = 5.07, *SD* = 1.46). Followed by cost-effectiveness described in concrete terms (mean acceptance rating = 4.89, *SD* = 1.55), and patient waiting time (mean acceptance rating = 4.38, *SD* = 1.68). The priority setting principle that received the lowest level of support was to give patients in need an equal chance of getting treatment by using a random selection principle (mean acceptance rating = 2.21, *SD* = 1.44). Acceptance was also generally low when subjects were asked if opportunity costs should be considered when setting priorities (mean acceptance rating = 2.77, *SD* = 1.69). The acceptance toward the three general priority setting principles from the Swedish ethical platform (average acceptance rating = 5.41, *SD* = 0.93) where generally higher than for the more concrete principles in the context of COVID-19 (average acceptance rating = 3.78, *SD* = 0.85, *t*_(1, 547)_ = −51.86, *p* < 0.001). Among the general priority setting principles, the human dignity principle, which prescribes equal access to care regardless of personal characteristics, was the most accepted (average acceptance rating = 5.85, *SD* = 1.50). Followed by the needs- and solidarity principle, which prescribes that healthcare's resources should be allocated to those with the highest needs, which had a mean acceptance rating of 5.52 *(SD* = 1.35). The cost-effectiveness principle which prescribes a reasonable relation between costs and effects was the general principle with the lowest level of acceptance (average acceptance rating = 4.86, *SD* = 1.56). Thus, the level acceptance among the three general principles followed the lexical order in which they are presented in the Swedish ethical platform. The mean levels of acceptance were significantly different for all the three general priority-setting principles (*p* < 0.001).

As illustrated in Figure 1, there is a significant difference in acceptance when cost-effectiveness was presented as an abstract principle in the context of COVID-19 (Item 1.6) compared to when cost-effectiveness was presented as a concrete principle (Item 1.7). Mean acceptance rating was 1.25 scale points higher when cost-effectiveness was presented as a concrete principle (Mean acceptance rating cost-effectiveness_abstract_ = 3.64 *SD* = 1.76, Mean acceptance rating cost-effectiveness_concrete_ = 4.89 *SD* = 1.55, *t*__(_1, 546)_=28.72, *p* < 0.001). This difference was robust when adjusting for age, gender, and education in a regression framework (Supplementary Table S1 in Supplementary Materials). Figure 1 also shows a general difference in attitudes toward priority setting principles when described as abstract general principles compared when described as concrete principles. The general and more abstract principles from the Swedish ethical platform were on average judged 1.62 points more acceptable than the more concrete principles for prioritizing patients during COVID-19 (mean acceptance rating general abstract principles = 5.41 *SD* = 0.93, Mean acceptance rating concrete COVID-19 principles = 3.79 *SD* = 0.84, *t*__(_1, 547)_ = 51.86, *p* < 0.001). This result was robust also when adjusting for age, gender, and education in a regression framework (Supplementary Table S1 in Supplementary Materials).

**Figure 2:**
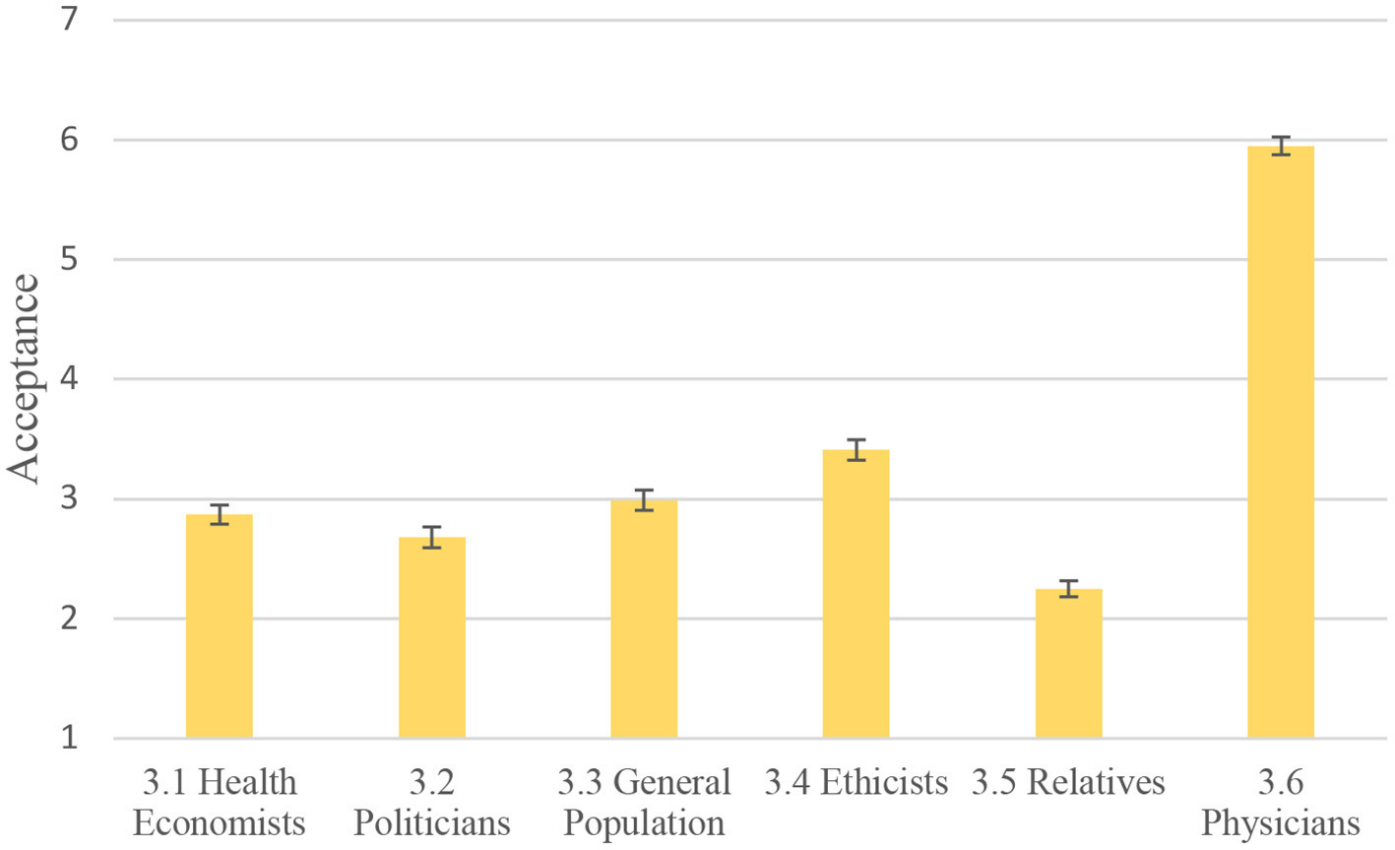
Attitudes toward whom should have primary responsibility for priority setting decisions during COVID-19. The figure shows mean ratings of acceptance with bars for 95% confidence intervals. 1 = lowest acceptance, 7 = highest acceptance.

Turning to the question of who should be responsible for making priority-setting decisions during COVID-19, Figure 2 illustrates that participants thought physicians should be primarily responsible for priority setting and allocation of scarce resources in healthcare. By a large margin physicians received the highest support for having the responsibility for deciding how scarce resources should be allocated (mean acceptance rating = 5.95, *SD* = 1.14). Ethicists followed with a mean support rating of 3.41*(SD* = 1.71), while politicians and health economists both received a mean acceptance rating below 3 on the presented 1–7 Likert scale.

## Discussion

Who shall live and who shall die? The COVID-19 pandemic forced decision makers at all levels of health care systems across the world to think hard about priority setting dilemmas that were previously most typically encountered only in hypothetical trolley problems. In this survey, we assessed public attitudes (in a Swedish sample) toward different principles to base priority-setting decisions on during the pandemic and in general. Our results show that the priority setting principles that received the highest support during the pandemic were to prioritize patients based on the severity of the condition and to allocate resources to patients who would benefit the most from treatment. Principles related to patients age and individual responsibility for own medical conditions received less support among respondents. Thus, we see a relative low support for “libertarian” priority setting principles. Our findings are largely consistent with the values identified as broadly supported in a group discussion study (*n* = 60) performed in the UK pre-COVID: (1) a broad “rule of rescue” that gives priority to those in immediate need, (2) health maximization and (3) equalization of lifetime health (27). They are also largely consistent with van Exel et al. (25) who used a Q Methodology (*n* = 294) to elicit societal views regarding appropriate principles in priority setting across 10 European countries and observed that expressed viewpoints in general gave little support to libertarian views, while considerable support for egalitarian ones. Although our survey illuminates different levels of acceptance for normative principles in priority setting it is important to remember that no single priority setting principle can underpin priority setting. Priority setting will always involve a plurality of normative values which may carry different weight in different contexts.

A main aim of this study was to compare attitudes toward cost-effectiveness and other values in healthcare priority setting presented at different levels of abstraction. Our results show that people were more supportive of cost-effectiveness as a basis for priority setting when presented in concrete terms related to health maximization rather than in general terms. Thus, our findings are in line with previous studies suggesting that people become more oriented toward efficiency concerns when presented with specific policy problems, while they gravitate toward equality concerns when policies on resource allocation are described in more general terms. Besides values related to cost-effectiveness, we see a general effect that respondents have higher acceptance toward abstract general priority-setting principles, such as those included in the Swedish ethical platform. As argued elsewhere (28), these principles are inherently vague and give little guidance in concrete situations where trade-offs between efficiency and other ethical values need to be made.

Finally, our results reveal a high level of support for physicians making priority setting decisions during the COVID-19 pandemic, while being much less supportive of health economists and politicians playing an active role in deciding how scarce resources should be allocated. This is perhaps not surprising but underscore the importance of involving medical professionals in the priority setting process to ensure public legitimacy during a global public health crisis. Moreover, it suggests a commonly held view that health care priority setting should be based on value-free medical facts rather than normative values. Medical facts are obviously relevant to any health care priority setting decision but choosing to remain ignorant about the normative values will inevitably increase the risk of implementing decisions and policies that run afoul of the overarching goals of any health care system. It should be noted that we asked participants explicitly about responsibility for allocating scarce resources during COVID-19. Thus, we do not know if this support for physicians compared to other groups of medical decision makers is equally strong in non-pandemic times where the feeling of emergency is less present.

There are some limitations that should be acknowledged. The survey was not designed as a strict experiment and does not keep everything identical across statements except for the level of abstraction. Thus, we cannot rule out other differences between principles influence the results. Most importantly, the concrete principles in the survey concerned COVID-19 at the intensive care, while the more abstract principles of the Swedish ethical platform were presented for health care in general and not specifically related to COVID-19. Thus, our findings are not a strict test of the effect of level of abstraction on attitudes toward priority setting principles and should therefore be taken as suggestive. However, given alignment between our findings and theoretical predictions, the results found here warrant further consideration and testing. Construal Level Theory (14) predicts that higher-level abstraction prompts people to focus on central values instead of practical concerns. Our finding that cost-effectiveness was more supported when presented in specific terms fits well with this prediction.

Previous studies have shown that emotional valence often leads to compassion fade in helping behavior, making people insensitive to magnitude of a policy problem (13, 29, 30). Our results add to this literature by suggesting that attitudes toward how scarce health care resources should be allocated are likely to be sensitive also to at what level of abstraction a priority-setting principle is described. Cost-effectiveness often is presented as a practical concern rather than a profound moral value. Our results suggest that this could be a key reason for why the health economic endeavor to maximize health within a budget does not strike a chord among the public when it comes to priority setting at the general level.

## Data Availability Statement

The raw data supporting the conclusions of this article will be made available by the authors, without undue reservation.

## Ethics Statement

Ethical review and approval was not required for the study on human participants in accordance with the local legislation and institutional requirements. The participants provided their written informed consent to participate in this study.

## Author Contributions

GT designed the study and drafted the manuscript together with LS. LS conducted the data analyses. Both authors contributed to the article and approved the submitted version.

## Funding

The study was funded by the *Swedish Research Council for Health, Working Life and Welfare*–FORTE 2019-01101. The funders had no role in study design, or preparation of the manuscript.

## Conflict of Interest

The authors declare that the research was conducted in the absence of any commercial or financial relationships that could be construed as a potential conflict of interest.

## Publisher's Note

All claims expressed in this article are solely those of the authors and do not necessarily represent those of their affiliated organizations, or those of the publisher, the editors and the reviewers. Any product that may be evaluated in this article, or claim that may be made by its manufacturer, is not guaranteed or endorsed by the publisher.
