# Supplementary material for: Public Attitudes Toward Priority Setting Principles in Health Care During COVID-19
Source: Front Health Serv. 2022 May 13;2:886508. doi: 10.3389/frhs.2022.886508 (PMC10012618; doi:10.3389/frhs.2022.886508)
Supplement: Supplementary file 1 [file Table_1.DOCX]

Supplementary materials - Public attitudes toward priority setting principles in health care

| Table S1 - Attitudes towards specific and general principles for health care priority setting | | |
| --- | --- | --- |
| Dependent Variable | COVID-19 Principles | General Principles |
|  | Coefficient (SE) | Coefficient (SE) |
| Age | -0,007* (0,003) | -0,001 (0,002) |
| Male | -0,101 (0,092) | 0,399*** (0,066) |
| Education |  |  |
| At most Primary School | 0,201 (0,172) | 0,144 (0,123) |
| Secondary School | 0,142 (0,120) | 0,141 (0,086) |
| Higher Education (<2 years) | 0,097 (0,109) | 0,127 (0,078) |
| Constant | 1,631*** (0,187) | -1,914*** (0,134) |
| Observations | 1515 | 1521 |

Note: All regressions are OLS. Concrete Principles was measured as the difference between the subjects’ acceptance towards prioritizing intensive care during COVID-19 based on either prioritizing treatments with a high effect with a low effort (concrete principle) or where treatment is cost-effective (abstract principle). Thus, a positive value indicates a higher acceptance towards cost-effectiveness when it is expressed more concrete (item 1.7) over as a more abstract principle (item 1.6). General Principle was measured as the difference between the subjects’ mean acceptance towards prioritizing intensive care during COVID-19 based on different concrete factors and the average acceptance for the principles from Sweden’s ethical platform in priority setting. Thus, a positive value indicates a higher preference towards prioritizing based on general terms (average item 1.1 to 1.10) than general principles (average item 2.1 to 2.3). Higher ≥ 3 years is the reference educational level. *** p < 0.001, ** p < 0.01, * p < 0.05

S2 Transcript of Survey

[Introduction]

We would like to ask you to answer some questions about the political situation in Sweden and thereby support our research project. The survey only takes about 5 minutes. There are no right or wrong answers, just answer in a way that reflects your own personal opinions. Thank you so much for your participation!

[i) Priority-setting principles of intense care units during COVID-19]

In connection with the Covid-19 pandemic, health care and society are faced with difficult priorities on how scarce health care resources should be distributed. This raises questions about how these priorities should take place if everyone who needs them cannot have access to intensive care units. Indicate to what extent you agree with the statements below for how the scarce resources of healthcare should be distributed.

|  | Strongly disagree | Disagree | Somewhat disagree | Neither agree nor disagree | Somewhat agree | Agree | Strongly agree |
| --- | --- | --- | --- | --- | --- | --- | --- |
| Higher priority should be given to infected patients who have followed the Public Health Agency of Sweden’s recommendations and acted responsibly to avoid the virus. |  |  |  |  |  |  |  |
| Higher priority should be given to infected patients who have responsibilities to take care of children or other relatives. |  |  |  |  |  |  |  |
| Higher priority should be given to infected patients who have an important societal function. |  |  |  |  |  |  |  |
| Higher priority should be given to younger infected patients. |  |  |  |  |  |  |  |
| Higher priority should be given to infected patients with worse health conditions. |  |  |  |  |  |  |  |
| Higher priority should be given to infected patients where treatment is cost-effective |  |  |  |  |  |  |  |
| Higher priority should be given to infected patients where a limited effort is expected to have a large effect |  |  |  |  |  |  |  |
| If the resources are not sufficient to give everyone treatments it is reasonable to use a lottery to decide who should get treatment |  |  |  |  |  |  |  |
| If the resources are not sufficient to give everyone treatments it is reasonable to let the patient who has waited longest get treatment. |  |  |  |  |  |  |  |
| When you give treatment to an infected patient you should always consider what you instead could have used the resources for. |  |  |  |  |  |  |  |

[ii) General priority setting principles for healthcare]

In Sweden, the Riksdag has decided on a so-called ethical platform consisting of three ethical principles that will support and provide guidance in prioritizing scarce healthcare resources.

Indicate to what extent you agree with the statements on how the scarce resources of healthcare should be distributed as described below

|  | Strongly disagree | Disagree | Somewhat disagree | Neither agree nor disagree | Somewhat agree | Agree | Strongly agree |
| --- | --- | --- | --- | --- | --- | --- | --- |
| Human dignity principle: All humans have the same value and the same right to care independent of personal traits and function in society. |  |  |  |  |  |  |  |
| The needs- and solidarity principle: More of the healthcare’s resources should be spent on the person or the organization which have the greatest needs. |  |  |  |  |  |  |  |
| The cost-effectiveness principle: In choices between different areas of operations or measures should a reasonable relation between costs and effects, measured in improved health or increased quality of life, be sought. |  |  |  |  |  |  |  |

[iii) Decision-maker for the healthcare’s prioritizations during COVID-19]

Indicate the extent to which you agree with the following positions on how the scarce resources of healthcare under Covid 19 should be distributed.

|  | Strongly disagree | Disagree | Somewhat disagree | Neither agree nor disagree | Somewhat agree | Agree | Strongly agree |
| --- | --- | --- | --- | --- | --- | --- | --- |
| Decisions of how scarce resources in the healthcare should be allocated should primarily be decided by health economists. |  |  |  |  |  |  |  |
| Decisions of how scarce resources in the healthcare should be allocated should primarily be decided by politicians. |  |  |  |  |  |  |  |
| Decisions of how scarce resources in the healthcare should be allocated should primarily be decided by the general population. |  |  |  |  |  |  |  |
| Decisions of how scarce resources in the healthcare should be allocated should primarily be decided by ethicists |  |  |  |  |  |  |  |
| Decisions of how scarce resources in the healthcare should be allocated should primarily be decided by relatives |  |  |  |  |  |  |  |
| Decisions of how scarce resources in the healthcare should be allocated should primarily be decided by physicians and others within the medical profession. |  |  |  |  |  |  |  |

[Gender]

What is your gender?

- Male
- Female
- Other ________________________________________________

[Age]

How old are you?

________________________________________________________________

[Education]

What’s your highest completed education?

- Lack formal education
- *Volksschule*, 6 years
- *Realschule*, faculty school or girl school
- Primary school, 9 years
- Secondary school, matriculation examination
- Additional education after secondary school (e.g., qualified vocational training)
- Health school, social collage (older education)
- Bachelor’s degree or college degree/college engineer
- Master’s degree, civil engineer, or other similar longer education
- Postgraduate education (licentiate or PhD)
